# The Identification and Management of Subthreshold Depression and Anxiety in Primary Care for People With Long-Term Conditions

**DOI:** 10.1155/da/9497509

**Published:** 2025-03-06

**Authors:** Patrick Cabasag, Frederick Sundram, Amy Chan, Kebede Beyene, Lauren Shepherd, Jeff Harrison

**Affiliations:** ^1^School of Pharmacy, Faculty of Medical and Health Sciences, The University of Auckland, Auckland, New Zealand; ^2^Department of Psychological Medicine, Faculty of Medical and Health Sciences, The University of Auckland, Auckland, New Zealand; ^3^Department of Pharmaceutical and Administrative Sciences, University of Health Sciences and Pharmacy in St. Louis, St. Louis, Missouri, USA; ^4^School of Psychology, Faculty of Science, The University of Auckland, Auckland, New Zealand; ^5^Department of Physiology, Faculty of Medical and Health Sciences, The University of Auckland, Auckland, New Zealand

**Keywords:** identification, long-term conditions, management, primary care, subthreshold anxiety, subthreshold depression

## Abstract

**Background:**

Subthreshold depression (sDep) and anxiety (sAnx) are common conditions and are associated with significant suffering, impaired functioning, increased healthcare utilisation and economic costs. Furthermore, they are risk factors for crossing the clinical threshold and developing mental health disorders. Subthreshold conditions are associated with long-term conditions (LTCs). This scoping review aimed to explore the identification and management of sDep and sAnx in primary care for patients with LTCs.

**Methods:**

We conducted a scoping review, following the Joanna Briggs Institute (JBI) Manual for Evidence Synthesis. Medline, PsycInfo, CINAHL and International Pharmaceutical Abstracts were searched for articles prior to September 2023. We included studies written in English that were conducted among the adult population. All studies that aimed to identify and manage sDep and anxiety in patients with LTC in primary care have been included.

**Results:**

Thirty-three articles were included in this scoping review, of which seven studies incorporated an intervention component for sDep and sAnx in patients with LTCs. A variety of definitions and screening tools were used to identify sDep and sAnx. Problem-solving therapy (PST) and behavioural activation (BA) were the most common intervention components and showed promising results.

**Limitations:**

We excluded studies that did not explicitly state the terms 'subthreshold', 'subclinical' or 'subsyndromal' depression or anxiety which may be relevant.

**Conclusion:**

There is currently limited evidence regarding the identification and management of sDep and sAnx in patients with LTCs, warranting further research.

## 1. Introduction

Subthreshold depression (sDep) and anxiety (sAnx) are prevalent in the general population and represent conditions in which people experience signs and symptoms that are significant enough to affect their lives, but do not meet the threshold for a clinical diagnosis [1, 2]. The lifetime prevalence of sDep is estimated to be between 10% and 24% [3–5] and the 3-year prevalence of sAnx is estimated to be around 11.4% [6].

sDep and sAnx cause significant suffering and functional impairment among individuals [2, 7, 8]. These conditions also have a considerable impact on the quality of life of individuals, resulting in increased healthcare utilisation and considerable economic costs [9–11]. Research has shown that people experiencing subthreshold conditions suffer from distress and impaired functioning comparable to those with diagnosed mental health disorders [4]. Importantly, sDep and sAnx are risk factors for progression into their respective clinical disorder and other mental disorders in 14%–35% of people when assessed between 3 and 25 years later [12].

Like their clinical 'threshold' equivalents, sDep and sAnx are linked to long-term conditions (LTCs). Around one third of people with pre-existing LTCs experience symptoms of depression [13]. Studies have shown that sDep can exacerbate LTCs, such as diabetes and cardiovascular disease [14]. Although few studies have explored the relationship between sAnx and LTCs, they have shown that sAnx is associated with LTCs such as cardiovascular disease and chronic obstructive pulmonary disease (COPD) [15]. These studies demonstrate the bidirectional relationship between mental health conditions and long-term physical conditions.

While there have been studies on sDep, and to a lesser extent sAnx, in the general population, little is known regarding the identification and management of these conditions in patients with LTCs in the primary care setting. Individuals with these conditions are less likely to present in specialist settings compared to primary care [16]. Therefore, identifying and managing sDep and sAnx in primary care settings is critical for early detection, timely intervention and potentially reducing the need for pharmacological treatment and specialist services. The existing literature on comorbid physical and mental health problems predominantly focuses on clinical depression and anxiety. However, sDep and sAnx are arguably just as important in LTC patients and contribute to a similar degree of impairment in quality of life and functional status [4]. Despite this, no review has explored this area. Given the lack of previous reviews and the likely breadth of literature, a scoping review methodology was undertaken. The aim of this scoping review is to synthesise evidence on the definitions, identification and management of sDep and sAnx in adults with LTCs in primary care.

## 2. Methods

This scoping review follows the methods from the Joanna Briggs Institute (JBI) Manual for Evidence Synthesis [17], which are based on the works of Arksey and O'Malley [18] and Levac, Colquhoun and O'Brien [19].

The study protocol is available on the OSF registries (https://doi.org/10.17605/OSF.IO/457DB).

### 2.1. Search Strategy

A search was conducted for published literature in Medline, PsycInfo, CINAHL Complete and International Pharmaceutical Abstracts. Studies published until September 2023 were included, with no lower date limit applied. Keyword search terms and MeSH headings were used to conduct the search. However, LTCs were intentionally excluded as a keyword or MeSH heading because of the scarcity of relevant studies specifically labelled under this term. Instead, studies related to LTCs were identified through manual screening. No further restrictions were applied to the search process. Reference lists were hand-searched for additional relevant studies. Grey literature was identified through targeted searches of dissertations/theses and conference abstracts. An example search strategy for the PsycInfo database can be found in *Supporting Information 1*.

### 2.2. Inclusion and Exclusion Criteria

This scoping review considered a broad range of published literature, including systematic reviews, observational studies, clinical trials, case studies, research protocols and any other relevant studies. We also considered grey literature, dissertations, discussion papers, government reports, expert opinions, position papers and other texts as they could potentially be relevant to the review's objectives.

Studies were eligible for inclusion if they included adults (≥18 years) with at least one LTC. LTCs were defined as any ongoing, recurring or long-term physical conditions that can significantly impact people's lives, including but not limited to, diabetes, cardiovascular diseases, respiratory diseases and cancers. Only studies conducted in primary care settings were included, defined as settings with no/limited specialist involvement. sDep and sAnx (or similar terms—subclinical or subsyndromal) were defined as signs and symptoms of depression and anxiety that do not meet the diagnostic criteria for a clinical diagnosis of depression and anxiety. Articles that explicitly referred to subthreshold, subclinical or subsyndromal depression and anxiety, even when no diagnostic interview had been conducted were included. This includes also studies that reported the non-pharmacological interventions for sDep and sAnx, and studies involving the identification of sDep and sAnx, regardless of whether it was the main study aim. Any studies that involved the identification of sDep/sAnx were included, irrespective of whether the study included an intervention component.

Any depressive and anxiety disorders meeting the diagnostic criteria and any other terms not stating subthreshold, subclinical or subsyndromal were excluded from the review.

### 2.3. Study/Source of Evidence Selection

Following the literature search, all identified articles from the different databases were uploaded into a reference management software (Endnote). Rayyan software [20] was used to screen studies and duplicates were removed using this software.

Two of the authors (Patrick Cabasag and Jeff Harrison) independently screened study titles and abstracts during the initial search. The inclusion criteria related to LTCs and adults aged 18 years and above were not applied during preliminary screening, as these characteristics were challenging to discern from titles and abstracts alone. Following the initial screening, Patrick Cabasag and Jeff Harrison reviewed the full-text articles to ensure they met all the specified inclusion criteria. Any differences in opinions between the authors were resolved through consensus.

Pilot testing of 25 randomly selected articles was conducted at the beginning of the study for quality assurance, as per JBI recommendation [17]. Patrick Cabasag, Jeff Harrison and Lauren Shepherd independently screened the titles and abstracts of these 25 articles and included studies based on the predefined inclusion and exclusion criteria. Patrick Cabasag, Jeff Harrison and Lauren Shepherd discussed any discrepancies and updated the inclusion/exclusion criteria as needed. Title and abstract screening only continued once a 75% or greater agreement was achieved.

### 2.4. Data Extraction/Charting

Data relevant to the review objectives were collected from the included studies using a data extraction form (*Supporting Information 2*). The extracted data included the title, authors, year of publication, objectives, study design and population, condition(s) studied, identification/screening methods, description of treatments/interventions and other key findings. For studies without an intervention component, the study designs were not extracted as they were not relevant to our review's objectives, but the definitions and instruments used were still considered relevant.

Following guidance from the JBI Manual [17], Patrick Cabasag and Lauren Shepherd piloted the data extraction form using three methodologically varied articles from the included set to confirm its suitability for capturing all relevant information for this scoping review. Any necessary modifications to the form were made through discussions between Patrick Cabasag and Lauren Shepherd. Data extraction was independently carried out by both authors (Patrick Cabasag and Lauren Shepherd), with a subsequent check performed by Patrick Cabasag.

## 3. Results

**Figure 1:**
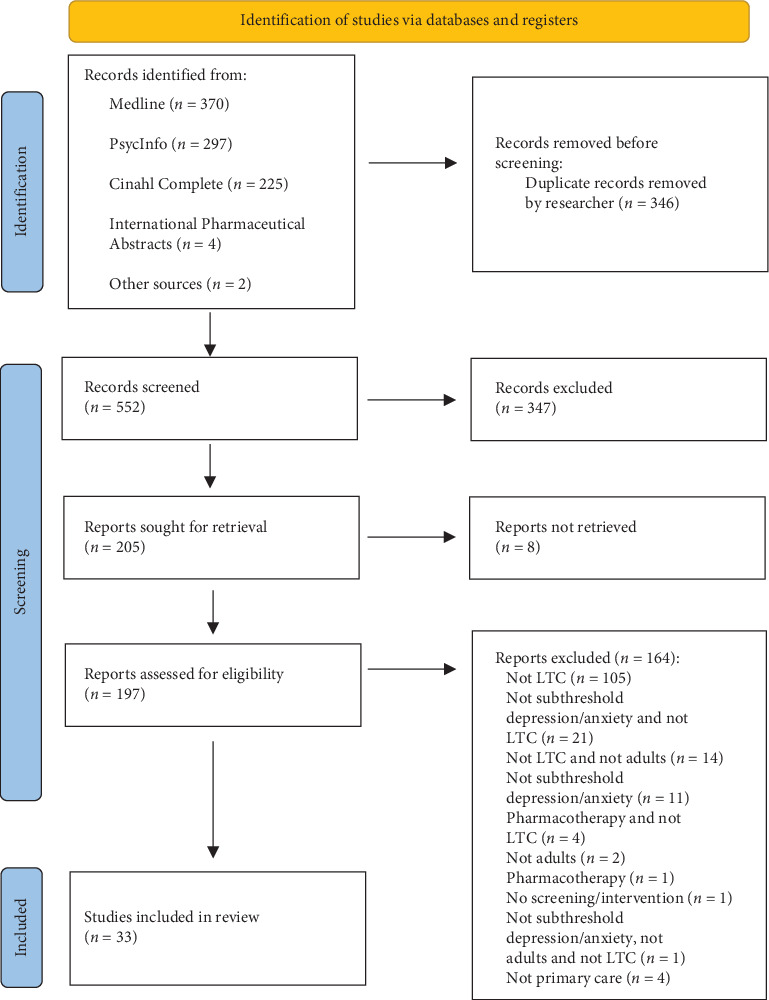
PRISMA search.

In the initial search, 898 articles were retrieved. Following the removal of duplicates, 552 articles underwent title/abstract screening. This narrowed down the selection to 197 articles for full-text evaluation, out of which 33 were considered for in-depth review. From these 33 articles, 17 unique studies were included in the final review. A detailed breakdown of exclusions post full-text screening is provided in Figure 1.

### 3.1. Characteristics of Included Studies

Studies included in the review were mostly related to sDep, with only four studies related to sAnx [21–24].

#### 3.1.1. Non-Intervention Studies

In studies without an intervention component, many did not primarily focus on LTC patients with sDep and sAnx. Nevertheless, these studies identified and defined sDep and sAnx amongst LTC patients. The criteria and instruments used provided clarity on how the literature has defined and identified sDep and sAnx.

Geographically, five studies were conducted in the USA [25–29], three in Europe [30–32] and the remaining two studies were from Brazil [33] and Canada [23]. Of these, nine studies focused on specific chronic conditions [23, 25–27, 29–33], while one covered a range of LTCs without restricting to specific condition(s) [28]. The LTCs included diabetes [25, 26, 31], cardiovascular disease [26, 29, 33], painful chronic conditions [23], COPD [30], osteoarthritis [27] and pituitary adenomas [32].

#### 3.1.2. Intervention Studies

Among the studies that incorporated an intervention component, six were fully randomised controlled trials (RCTs) [21, 22, 24, 34–36] and another six were intervention protocols [37–42]. The remaining studies included cost-effectiveness studies [34, 43–45], 2-year effectiveness studies [46, 47] and feasibility studies [48].

The majority of the intervention studies (*n* = 6) were conducted in Europe [22, 24, 34–36, 48], with one study conducted in India [21].

Three studies focused on specific chronic conditions [24, 35, 36], while the other four examined a broader spectrum of LTCs [21, 22, 34, 48]. The LTCs investigated included visual impairment [24], diabetes [35, 36] and cardiovascular disease [35].

A summary of the included studies can be found in Tables 1 and 2.

### 3.2. Identification of sDep and sAnx

A summary of the conceptualised definitions of sDep and sAnx, as stated in the introduction of the included studies, is presented in Table 3.

Depressive and anxiety symptoms were primarily identified using various screening tools. In some studies, diagnostic interviews were used to exclude a mental health disorder diagnosis. The patient health questionnaire (PHQ) was the predominant screening tool, utilised in five studies [25, 27, 33, 35, 36]. Other tools employed included the Centre for Epidemiologic Studies Depression Scale (CES-D) [22, 24], hospital anxiety and depression scale (HADS) [24, 30], Whooley questionnaire[34], Beck depression inventory (BDI) [32], global assessment score (GAS) [28], and the General Health Questionnaire (GHQ) [21].

Regarding methodological details, six studies relied on self-report measures, using specific scores to determine if participants had sDep and sAnx [25, 27, 30–33]. Eleven studies used diagnostic interviews to identify sDep and sAnx and exclude major depression [21–24, 26, 28, 29, 34–36, 48]. Of these, eight administered the diagnostic interview after the complementary use of a screening tool [21, 22, 24, 26, 28, 34–36]. Most studies referred to the Diagnostic and Statistical Manual of Mental Disorders (DSM), with one study utilising the international classification of diseases (ICD) [31].

In studies incorporating an intervention component for sDep and sAnx, a diagnostic interview was consistently utilised. Most adopted the Mini International Neuropsychiatric interview (MINI) [21, 22, 24, 34, 35, 48], while one study used the Structured Clinical Interview for DSM Disorders (SCID) [36]. The purpose of these diagnostic interviews was to identify sDep and sAnx and exclude a clinical mental health disorder diagnosis. Moreover, five of these studies used diagnostic interviews according to DSM IV criteria [22, 24, 34–36]. The operational definitions and screening tools used across studies are presented in Tables 1 and 2.

### 3.3. Management of sDep and sAnx

Of the reviewed studies, seven incorporated an intervention component for managing sDep and sAnx. Notably, six of these studies were RCTs [21, 22, 24, 34–36], and one was a feasibility study [48]. The interventions included a collaborative care (CC) model, stepped-care models, psychotherapy interventions and other additional components. Regarding the psychological interventions, four studies used problem-solving therapy (PST) [21, 22, 24, 35], two used behavioural activation (BA) [34, 48] and one used psychoeducation and physical exercise [36].

Five of the studies [22, 24, 34–36] involved practitioners with mental health training, while two [21, 48] involved people with limited or no mental health training when delivering the psychological intervention. The practitioners with mental health backgrounds included nurses [22, 34, 35], psychologists [24, 34, 36], social workers [24] and occupational therapists [24]. In the two studies where health professionals with little or no mental health background carried out the intervention, community pharmacy staff [48] and lay health counsellors (LHCs) [21] (who were members of the local community with a bachelor's degree in a non-health related field) were responsible for delivering the interventions. Other health workers involved in additional components of the interventions included physiotherapists [36] and diabetologists [36].

In the two studies involving professionals with limited or no mental health background, specific training on intervention delivery was provided. Community pharmacy staff attended a 2-day workshop training, followed by a telephone-based competency assessment in the study by Chew-Graham et al.[48]. In comparison, Dias et al. [38] recruited LHCs using an interview process, in which LHCs were asked to role-play a basic aspect of the intervention. LHCs selected via interview went on to complete a 1-week training, covering all aspects of the intervention. After training was completed, LHCs were required to undertake a competency assessment, using the therapy quality assessment scale (TQS) [38].

A description of the intervention studies and their results can be found in Table 4.

#### 3.3.1. PST

PST is a cognitive behavioural intervention focused on practical skill building. The goal of PST is to teach active problem-solving skills as an alternative to avoidant coping [21, 22, 24, 35]. Four studies included PST as a part of their intervention [21, 22, 24, 35].

Three studies used the same stepped-care intervention approach [22, 24, 35]. The intervention included watchful waiting, where no active care was provided. After 3 months, if symptoms persisted (as determined by the PHQ-9 score), participants were moved on to the next step, which involved a written self-help course designed to reduce symptoms of depression in patients with a chronic medical condition. If PHQ-9 scores remained elevated, the next step was PST. Finally, if PST proved ineffective, the last step was referral to a general practitioner (GP). In contrast, Dias et al. [21] investigated a depression in late life (DIL) intervention that did not use a stepped-care approach and included several components, such as PST, brief behavioural treatment for insomnia, education in self-care for common medical disorders and assistance in accessing medical and social programmes.

The three studies using the stepped-care approach had a study duration of 12 months [22, 24, 35]. In comparison, the DIL intervention lasted 6–10 weeks, with booster sessions at seven and 10 months [21].

van der Aa et al. [24] and Dias et al. [49] demonstrated that their respective interventions were acceptable, through interviews and examination of dropout rates. With the exception of the study by Pols et al. [35], all PST interventions showed promising results in reducing symptoms of depression and anxiety and reducing the incidence of depressive and anxiety disorders [21, 22, 24]. Only two of the studies investigated the effects of their interventions over 2 years [46, 47]. van't Veer-Taelaar et al.[46] suggested that the intervention had sustained effects over 24 months. In contrast, a 2-year effectiveness study by Pols et al. [47] showed no statistically significant overall treatment effect over a 24-month period.

Last, each of the studies using the stepped-care approaches conducted an economic evaluation of their respective interventions [43–45]. van der Aa et al. [43] concluded that the stepped-care intervention was more effective and cost-saving than usual care, depending on the willingness to pay per disorder prevented. Similarly, Van't Veer-Taelaar et al. [45] suggested that their intervention provided depression and anxiety free survival years at an affordable cost. In contrast, van Dijk et al. [44] concluded that the stepped-care intervention was not more cost-effective than standard care, which was expected due to the limited effectiveness of the intervention, as noted by Pols et al. [35, 47]. No economic evaluation was conducted for the DIL intervention.

#### 3.3.2. BA

BA focuses on reducing avoidance of social interaction and rewarding activities while reintroducing positive reinforcement [34, 48]. It focuses on addressing behavioural deficits that impact healthy activities and encourages individuals to schedule such activities. One RCT [34] and one feasibility study [48] focused on BA as the main intervention component.

Both studies included several components in their respective interventions. Lewis at al. [34] investigated the use of CC and active surveillance for Screen-Positive elders with sDep (CASPER) intervention. The intervention included telephone support, symptom monitoring, active surveillance and low-intensity psychosocial intervention (in this case, BA). Chew-Graham et al. [48] investigated the feasibility and acceptability of an enhanced support intervention (ESI), which had components similar to the CASPER intervention, including symptom monitoring, proactive follow-up and BA. The ESI also included a signposting component for those at risk or if significant clinical deterioration was noted. A major difference between the two studies was that the ESI had a self-help focus approach, whereas the CASPER intervention did not.

The two studies also differed in the number of sessions delivered. The CASPER intervention provided eight weekly sessions to the participants [34], while Chew-Graham et al. [48] delivered four to six sessions. In terms of delivery mode, the CASPER intervention included one face-to-face session, followed by subsequent sessions conducted over the phone [34]. In comparison, the ESI study was more flexible, offering both face-to-face and telephone sessions [48]. Follow-up between these studies also differed: with CASPER intervention following participants for 12 months [34], whereas the ESI study had a shorter follow-up period of only 4 months [48].

The CASPER intervention proved to be clinically and cost-effective. Results indicated a statistically significant reduction in depressive symptoms, with lower PHQ-9 scores at 4 months of follow-up compared to usual care, and these differences persisted at 12 months. The intervention also reduced the proportion of participants developing depression compared to usual care [34]. Interestingly, a secondary analysis suggested that a minimum of five sessions is recommended for BA to be effective [50]. In contrast, Chew-Graham et al. [48] conducted only a feasibility study, so no conclusion could be drawn about the effectiveness of the intervention. However, the ESI was found to be acceptable to both participants and facilitators, and it was suggested that community pharmacies are an acceptable setting for offering a brief intervention to LTC patients at risk of depression. Additionally, the ESI and its patient supporting workbook were well-received by participants.

#### 3.3.3. Other Intervention Strategies

Lastly, Pibernik-Okanović et al. [36] compared psychoeducation, physical exercise and enhanced treatment as usual for treating subsyndromal depression in patients with type 2 diabetes. The enhanced care group involved a diabetologist addressing participants' diabetes-related concerns. This study differs from the other intervention studies by comparing three distinct interventions, each with different components. The psychoeducation and physical exercise interventions included six sessions, similar to other studies, while the enhanced treatment as usual intervention consisted only of one session. Additionally, this was the only study to use a small-group counselling approach, whereas the other interventions relied on individual techniques. In terms of effectiveness, Pibernik-Okanović et al. [36] suggested that even minimal intervention (as in enhanced intervention group) might be enough to treat subsyndromal depression, with participants in all three groups showing similar improvement in depressive symptoms.

## 4. Discussion

This scoping review is the first attempt to synthesise all the available evidence and identify gaps in research on the identification and management of sDep and sAnx amongst LTC patients in a primary care setting. Our findings show a significant knowledge gap in this area.

Few studies focus on the identification and management of sAnx in LTC patients compared to sDep. Similarly, fewer studies investigate sAnx than sDep in the general population. The lack of studies in sAnx may be due to two reasons. First, the focus on specific sAnx disorders, such as generalised anxiety disorder (GAD) and post-traumatic disorder (PTSD), has led to less research on sAnx as a broader term [6]. Second, the variability in definitions has made sAnx an ambiguous area. For some researchers, the term sAnx refers to the presence of some anxiety symptoms, while for others, it involves more specific criteria, such as the length of time anxiety symptoms have been present [15].

A broad range of definitions and screening tools were used to identify sDep and sAnx, highlighting the lack of consensus on defining these terms. While sDep and sAnx are generally defined as symptoms that do not meet diagnostic criteria, there is no detailed standardised definition (e.g., DSM or ICD) for these conditions [1, 2]. Consequently, these concepts are often defined operationally using scores on screening tools and diagnostic interviews to exclude clinical depression and anxiety. Due to this ambiguity, different cut-off scores were used for the same screening instruments across reviewed studies. Moreover, in the identified intervention studies, diagnostic interviews were typically used to distinguish subthreshold symptoms from diagnosed depression and anxiety. However, this approach may not be feasible in studies involving health professionals without prior mental health training.

Additionally, there is significant overlap between subthreshold conditions and terms used to describe other conditions, such as minor depression, mild depression/anxiety and depression and anxiety symptoms. Therefore, establishing a universal standardised definition of sDep and sAnx will be important for future research to enable comparisons across different studies. Although recent work by Volz et al. [51, 52] has explored the concepts of sDep and subsyndromal GAD, further research is needed. There is a need to clearly identify at which point symptoms of depression and anxiety are classified as 'significant', and when these symptoms are severe enough to affect daily functioning and warrant intervention. An agreement on the number, type and duration of symptoms will be helpful in achieving consensus on the definition of subthreshold terms.

Most of the RCTs in this scoping review show promising results regarding the effectiveness of the interventions, such as PST, BA and psychoeducation. These interventions were effective in reducing depressive and anxiety symptoms, as well as the incidence of major depression and anxiety disorders. This is consistent with the existing literature on the management of sDep and sAnx in the general population, which has also shown positive outcomes [53]. However, further research is needed to draw definitive conclusions.

The identification and management of LTC patients with sDep and sAnx is a critical area for primary care settings, such as general practice clinics and community pharmacies, where many people are likely to seek help as their first point of contact. Actively and routinely screening and managing subthreshold mental health conditions in LTC patients within primary care could be highly beneficial. Early detection and timely intervention may prevent progression to clinical thresholds, leading to broader benefits, such as reduced economic costs, decreased burden on the healthcare system and significant improvement in the quality of life for individuals [9–11]. Given the bidirectional relationship between physical and mental health, appropriately managing sDep and sAnx in LTC patients would be beneficial for both their mental and physical well-being.

The scoping review highlights the focus on primary care interventions for sDep and sAnx being delivered in GP clinics and practices, with few studies examining the role of alternative clinical settings, such as community pharmacies. Given the growing prevalence of mental illness, the increasing workload pressures on GPs and specialist mental health services, and the rapidly evolving role of pharmacists, investigating further the feasibility of their involvement in screening and delivering brief mental health interventions in a community pharmacy setting is warranted. Community pharmacies are widely distributed, accessible and the public's high level of trust [54] in pharmacists puts them in a prime position to help address the unmet needs of individuals with mental health conditions. LTC patients also have regular contact with their community pharmacy and community pharmacists are motivated and possess the mental health literacy to support those with mental health needs [55, 56]. However, there are several barriers that need to be considered when delivering interventions, such as BA in a community pharmacy setting. Chew-Graham et al. [48] identified factors, such as a lack of time and resources, and limited research experience among pharmacy staff as barriers to recruiting participants. While valuable lessons can be learned from the Chew-Graham et al. [48] study, further studies on sDep and sAnx are required in community pharmacy settings to understand and mitigate these barriers. Although no studies have yet demonstrated the effectiveness of pharmacist-led psychological interventions for sDep and sAnx, evidence shows that psychological interventions can be delivered effectively without requiring expensive or highly trained mental health professionals [57], who may be less accessible. The delivery of psychological interventions by non-mental health-trained professionals will increase access, while ensuring GPs and specialist mental health expertise is being used more effectively.

### 4.1. Strengths and Limitations

The strengths of this review include the broad inclusion and exclusion criteria, which allow for the comprehensive examination of the topic. Another strength is the robustness of the screening and data extraction process, which involved two researchers independently screening both titles/abstracts and full-text articles, as well as conducting data extraction. Pilot testing during the screening phase and the use of a data extraction form was also incorporated as quality assurance steps to increase the robustness of the process. However, a possible limitation of this review was the exclusion of terms such as minor depression and mild depression/anxiety, which may have led to the omission of relevant studies. While these terms could be considered part of the 'subthreshold' category, they are sometimes described separately in the literature. The heterogeneity of the studies made it difficult to assess and compare their quality. To avoid confusion, we have solely focused on subthreshold, subclinical or subsyndromal studies for this scoping review. Last, a limitation of the scoping review methodology is that it does not involve a thorough assessment of the risk of bias or the quality of the studies. In scoping reviews, articles are not typically critically appraised [58]. However, the findings from this scoping review have informed the scope and nature of the research and lay the foundation for a future systematic review when sufficient studies are available.

### 4.2. Implications of the Findings for Practice and Research

Further investigation is essential to provide healthcare practitioners with clear guidelines on managing LTC patients with sDep and sAnx. While this scoping review sought to assess the feasibility of a systematic review, the current lack of evidence suggests it is premature for such an undertaking due to the limited and heterogeneous studies. More RCTs in primary care would enrich the literature and pave the way for a subsequent systematic review. Most RCTs in this review indicated potential benefits in treating LTC patients with sDep and sAnx, but high-quality RCTs are imperative to determine the true impact of these interventions.

Studies examining the role of health professionals in the screening and management of sDep and sAnx in primary care are also needed. Given the challenges in implementing mental health interventions, qualitative and codesign studies would be beneficial to develop interventions that meet the needs of consumers and health professionals. This could result in a more acceptable and effective intervention for all involved.

Based on our scoping review and prior studies, people with LTCs are more likely to experience subthreshold as well as clinical depression and anxiety [59]. We have formulated some recommendations to improve the care and management of sDep and sAnx (Table 5), which we describe further below. Screening for both depression and anxiety, rather than either condition in isolation, would be sensible given the strong overlap of the two conditions in clinical settings [60]. For example, it would be feasible to screen LTC patients annually for depression and anxiety alongside routine physical health assessments. This is a practical and reasonable timeframe to monitor any changes in the mental health of LTC patients.

Balancing brevity, reliability and validity, the PHQ-9 and GAD-7 questionnaires would be appropriate tools for screening LTC patients with sDep and sAnx, respectively, in primary care. When using a threshold of a score of 10 or more, both tools have good specificity and sensitivity for clinical depression and GAD [61, 62]. PHQ-9 and GAD-7 scores between 0 and 4 indicate minimal to no symptoms, while scores between 5 and 9 could be used as a measure for sDep and sAnx.

**Figure 2:**
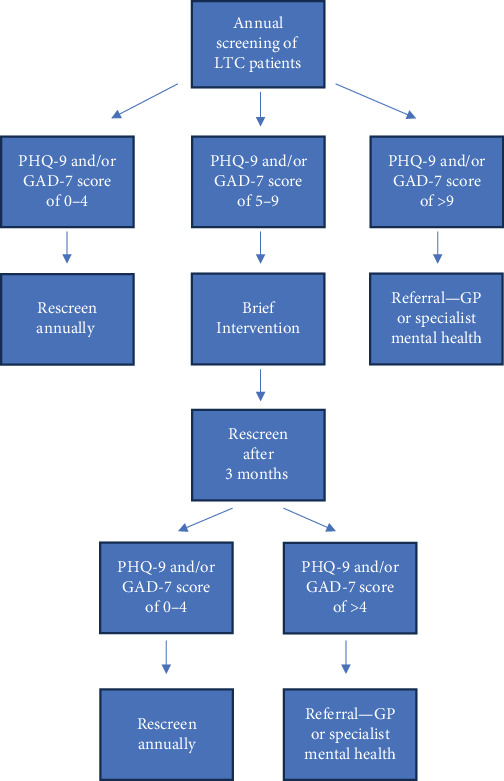
Possible screening and intervention pathway in a primary care setting for long-term condition (LTC) patients with depression and/or anxiety.

Appropriate management for LTC patients with sDep and sAnx would involve delivering brief interventions in a stepped-care approach. Self-help or guided self-help low-intensity psychological interventions, such as BA and PST would be potential options for managing sDep and sAnx. Other intervention options, such as psychoeducation and general lifestyle counselling may also be appropriate. Referral to GPs or specialists is necessary when the above treatments are not sufficient or when depressive and anxiety symptoms progress to more severe presentations. A possible intervention pathway is shown in Figure 2.

Non-mental health-trained professionals have the potential to deliver brief interventions with appropriate training. Studies in this review have shown that non-mental health-trained professionals can effectively deliver brief interventions, such as BA and PST [21, 48]. Therefore, this area is suitable for a range of health professionals, as it is less complex than managing clinically diagnosed disorders. Health professionals, such as pharmacists, can significantly contribute to easing the burden on GPs and mental health services, while maximising the reach of potential mental health interventions. While there is potential for non-mental health-trained professionals to deliver interventions, further exploration is needed in this area as there are several aspects to the process, and the screening process may need assessment by a trained health professional.

**Figure 3:**
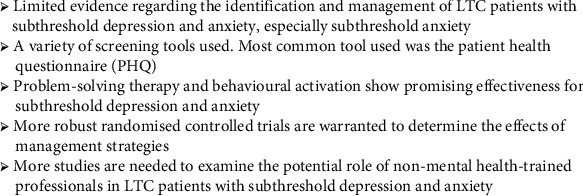
Take home messages.

The key take home messages of this review are summarised in Figure 3.

## 5. Conclusions

There is currently limited evidence regarding the identification and management of sDep and sAnx in LTC patients. A universal definition of sDep and sAnx is needed to avoid confusion and allow for comparisons between studies. While the intervention studies in this review show promising results, more robust, controlled trials are warranted to determine the effects of different intervention strategies in this area.

## Figures and Tables

**Table 1:** Summary of non-intervention studies.

Author and publication year	Condition	Population	Country	Operational definitions of sDep/sAnx	Diagnostic interview used
Czech et al. (2015) [25]	sDep	Type 2 diabetes	USA	A provisional diagnosis of sDep is measured using the PHQ-9, requiring 2–4 symptoms, including either depressed mood or anhedonia.	No
Blakemore et al. (2019) [30]	sDep	COPD	UK	The HADS is used to measure sDep, with scores of 4–7 indicating sDep.	No
Nakamura et al. (2022) [33]	sDep	Hypertension and diabetes	Brazil	A PHQ-9 score of 5–9, with at least one core depressive symptom (depressed mood or anhedonia), is used to define sDep.	No
Williams et al. (1995) [26]	sDep	Hypertension, cardiac disease, diabetes mellitus and/or arthritis	USA	Subsyndromal depression is defined as having depressed mood or anhedonia plus one to three additional symptoms from the DSM-III criteria.	Yes
Dirmaier et al. (2010) [31]	sDep	Type 2 diabetes	Germany	DSQ is used to assess sDep and diagnose depression according to ICD-10 criteria. 5–7 points on the DSQ indicated sDep.	No
Liu et al. (2019) [27]	sDep	Osteoarthritis	USA	A PHQ-8 score of 5–9 is used to identify subclinical depression.	No
Rapaport and Judd (1998) [28]	sDep	No specific chronic physical diseases	USA	sDep is defined as having ≥2 symptoms of depression, excluding the primary DSM-IV criteria of feeling sad, blue or anhedonic, along with GAS score of <75. The SCID and DIS are used to identify sDep.	Yes
Leistner et al. (2015) [32]	sDep	Pituitary adenomas	Germany	BDI, with a score of 10–18 for patients or DSQ, with a score of 5–7 for control subjects is used to define sDep.	No
Sherbourne et al. (1994) [29]	sDep	Hypertension	USA	DIS is used to screen for depression according to DSM-III criteria. Those who exceed the cut-off score on the brief depression symptom scale but did not meet criteria for a depressive disorder are classified as having sDep.	Yes
D'Aiuto et al. (2022) [23]	sAnx	Painful chronic conditions	Canada	A structured interview is used to assess anxiety symptoms in the last 6 months. The interview questions were based on the DSM-IV criteria. sAnx is defined as having at least one symptom of anxiety but not meeting the full DSM IV diagnostic criteria for any specific anxiety disorder.	Yes

Abbreviations: BDI, Beck depression inventory; COPD, chronic obstructive pulmonary disease; DIS, diagnostic interview schedule; DSM, Diagnostic and Statistical Manual of Mental Disorders; DSQ, depression screening questionnaire; GAS, global assessment scale; HADS, hospital anxiety and depression scale; ICD, international classification of diseases; PHQ-9, patient health questionnaire–9; sAnx, subthreshold anxiety; SCID, structured clinical interview for DSM-IV; sDep, subthreshold depression.

**Table 2:** Summary of intervention studies.

Author and publication year	Study type	Condition	Population	Country	Operational definition of sDep/sAnx	Diagnostic interview used
Lewis et al. (2017) [34]	RCT	sDep	No specific chronic physical diseases	UK	The two-item Whooley questionnaire identifies depressive symptoms when at least one of the two questions is answered positively. The MINI is then used to identify sDep (2–4 symptoms based on DSM IV) and to exclude MDD.	Yes
Chew-Graham et al. (2022) [48]	Feasibility study	sDep	No specific chronic physical diseases	UK	sDep is defined as having 2–4 symptoms of depression on the major depressive module of the MINI.	Yes
Pols et al. (2017) [35]	RCT	sDep	Type 2 diabetes mellitus and/or CHD	Netherlands	The PHQ-9 is used to identify patients with depressive symptoms, with scores of ≥6 indicating the presence of symptoms. The MINI is then used to apply the DSM-IV diagnostic criteria and rule out patients with clinical depression.	Yes
van der Aa et al. (2015) [24]	RCT	sDep and sAnx	Visual impairment	Netherlands and Belgium	The CES-D (scores of 16 or higher) and HADS-A (score of 8 or higher) are used to assess severity of depression and anxiety symptoms, respectively. The MINI and DSM-IV are used to rule out patients with clinical depressive and anxiety disorders.	Yes
van't Veer-Tazelaar et al. (2009) [22]	RCT	sDep and sAnx	No specific chronic physical diseases	Netherlands	The CES-D is used to screen for depressive symptoms, with scores 16 or greater indicating the presence of symptoms. The MINI is used to exclude major depressive and anxiety disorder according to DSM IV criteria.	Yes
Dias et al. (2019) [21]	RCT	sDep and sAnx	No specific chronic physical diseases	India	GHQ is used to screen for depression/anxiety symptoms, with scores >4 indicating the presence of symptoms. The MINI is used to exclude major depression and anxiety disorder.	Yes
Pibernik-Okanović et al. (2015) [36]	RCT	sDep	Type 2 diabetes	Croatia	A semi-structured phone interview is used to determine patients' eligibility, requiring at least one depressive symptom over the past month and the need for professional help, based on PHQ-2. The SCID for DSM IV Axis 1 Disorders is then used to exclude major depression and dysthymia.	Yes

Abbreviations: CES-D, The Centre for Epidemiologic Studies Depression Scale; CHD, coronary heart disease; DSM, Diagnostic and Statistical Manual of Mental Disorders; GHQ, General Health Questionnaire; HADS-A, hospital anxiety and depression scale-anxiety subscale; MDD, major depressive disorder; MINI, Mini International Neuropsychiatric interview; PHQ-2, patient health questionnaire–2; PHQ-9, patient health questionnaire-9; RCT, randomised controlled trial; sAnx, subthreshold anxiety; SCID, structured clinical interview for DSM-IV; sDep, subthreshold depression.

**Table 3:** Conceptualised definitions of subthreshold depression (sDep)/anxiety (sAnx).

Author and publication year	Condition	Conceptualised definition
Lewis et al. (2017) [34]	sDep	“Those who give positive responses to screening questions but who do not have sufficient levels of depressive symptoms to meet diagnostic criteria.”
Chew-Graham et al (2022) [48]	sDep	“Depressive symptoms which fall below criteria for a diagnosis of major depression.”
Nakamura et al. (2022) [33]	sDep	“Symptoms of insufficient intensity to meet criteria for depression diagnosis.”
Williams et al. (1995) [26]	Subsyndromal depression	“One to three Diagnostic and Statistical Manual of Mental Disorders (DSM)-III criteria for depression.”
Dias et al. (2017) [38]	sDep	“Some symptoms of a depressive disorder but do not meet diagnostic criteria for a depressive disorder.”
Pibernik-Okanović et al. (2015) [36]	Subsyndromal depression	“The presence of depressive symptoms that do not meet full diagnostic criteria for major depression or dysthymia.”
van't Veer-Tazelaar et al. (2009) [22]	sDep and sAnx	“Some symptoms that possibly foreshadow the onset of a mental disorder but who do not meet the DSM-IV diagnostic criteria.”
Sherbourne et al. (1994) [29]	sDep	“Individuals with depressive symptoms in the absence of depressive disorder.”
Pols et al. (2017) [35]	sDep	“Clinically relevant depressive symptoms without fulfilling the criteria for major depressive disorder (MDD).”
van der Aa et al. (2015) [24]	sDep and sAnx	“Clinically significant symptoms, but no actual disorder.”

**Table 4:** Description of intervention studies.

Author and publication year of main study	Main intervention component	Description of intervention	Providers received previous mental health training (yes/no)	Intervention providers	Results/outcomes
Lewis et al. (2017) [34]	BA	CC, in which telephone support and session-to-session symptom monitoring were provided. Participants were also offered BA. Setting: GP practices.	Yes	Nurses, psychologists	CC proved to be clinically and cost-effective. There were statistically significant differences in depressive symptoms. There was also a reduction in PHQ-9 scores at 4 months follow-up compared to usual care. It was suggested that five sessions is the minimum effective dose.
Chew-Graham et al. (2022) [48]	BA	ESI, which is based on BA within a CC framework. The intervention had four main elements: BA, proactive follow-up, symptom monitoring and signposting. Setting: Community pharmacies.	No	Community pharmacy staff	The ESI is acceptable to participants and facilitators. Community pharmacies were considered an acceptable setting to offer a brief intervention to LTC patients at risk of depression. The ESI and supporting patient workbook were acceptable to participants.
Pols et al. (2017) [35]	PST	SCP, with watchful waiting, guided self-help course, PST and referral as the intervention components. Setting: Primary care centres.	Yes	Nurses	The intervention was not more effective at preventing MDD compared to usual care. The intervention was not more effective than usual care in the prevention of MDD at 2 years of follow-up. SCP was not more cost-effective than usual care.
van der Aa et al. (2015) [24]	PST	SCP, with watchful waiting, guided self-help course, PST and referral as the intervention components. Setting: Outpatient low vision rehabilitation organisations.	Yes	Psychologists, social workers, occupational therapists	The intervention was acceptable. The stepped-care intervention was more dominant to usual care in terms of clinical effectiveness and cost savings. The SCP intervention significantly reduced the incidence of depression and anxiety over a 2-year period.
van't Veer-Tazelaar et al. (2009) [22]	PST	SCP, with watchful waiting, cognitive behaviour therapy-based bibliotherapy, brief cognitive behaviour therapy-based PST and referral as the intervention components. Setting: Primary care practices.	Yes	Nurses	The SCP intervention for prevention of depression and anxiety is effective in reducing the risk of clinical depression and anxiety. The intervention provided depression and anxiety-free survival years at an affordable cost.
Dias et al. (2019) [21]	PST	PST and BBTI interventions were provided. It also included an intervention to help in accessing government-sponsored medical and social programmes and education in self-management of common chronic diseases. Setting: Rural and urban primary care clinics.	No	LHCs	The study demonstrated that the intervention was feasible and acceptable. There was a reduced incidence of major depressive episodes compared to usual care.
Pibernik-Okanović et al. (2015) [36]	Psychoeducation, physical exercise and enhanced treatment as usual	Psychoeducation, physical exercise and enhanced care as usual as the interventions provided. Setting: University clinic.	Yes	Psychologists, physiotherapists, diabetologists	The study suggested that minimal intervention (enhanced intervention group) may be enough to treat subsyndromal depression, with participants in all three groups equally showing improvement in depressive symptoms.

Abbreviations: BA, behavioural activation; BBTI, brief behavioural treatment of insomnia; CC, collaborative care; ESI, enhanced support intervention; GP, general practitioner; LHCs, lay health counsellors; LTC, long-term condition; MDD, major depressive disorder; PHQ-9, patient health questionnaire-9; PST, problem-solving therapy; SCP, stepped-care program.

**Table 5:** Recommendations.

• Annual screening of long-term condition (LTC) patients for both depression and anxiety together in primary care by health professionals (e.g., community pharmacists, nurses, general practitioners (GPs)) trained in using screening tools and questionnaires.
• Patient health questionnaire-9 (PHQ-9) and generalised anxiety disorder (GAD)-7 screening tools can be used for identifying subthreshold depression (sDep) and subthreshold anxiety (sAnx; score of 5–9).
• Brief intervention for LTC patients with sDep and/or sAnx should include self-help or guided self-help low-intensity psychological interventions, such as behavioural activation (BA) or problem-solving therapy (PST), delivered by primary care health professionals (e.g., community pharmacists, nurses, GPs).
• Referral to a GP or psychiatrist is recommended if intervention is insufficient and/or if the patient progresses to clinical depression or anxiety after 3 months.
• Interventions can be delivered by non-mental health-trained professionals with appropriate training.
• Community pharmacists, who are accessible, trusted by the public and widely distributed, can play a key role in supporting mental health in primary care.

## Data Availability

The data that support the findings of this study are available from the corresponding author upon reasonable request.

## Supporting Information

Supporting Information 2 The data extraction form used to collect data relevant to the review questions and objectives is outlined in Online File 2.
