# Supplementary material for: The Identification and Management of Subthreshold Depression and Anxiety in Primary Care for People With Long-Term Conditions
Source: Depress Anxiety. 2025 Mar 6;2025:9497509. doi: 10.1155/da/9497509 (PMC11987070; doi:10.1155/da/9497509)
Supplement: Supporting Information 1 — An example search on the PsycInfo database is provided under Online File 1. [file 9497509.f1.docx]

**Online File 1 – Example PsycInfo Search**

Keyword search terms of "primary care" OR "primary healthcare" OR "primary health care" OR "general practic*" OR "gp" OR "gps" OR "primary medical care" OR "first line care" OR "general practitioner*" OR "family doctor*" OR "family physician*" OR "family practitioner*" OR "general physician*" AND "depress*" OR "anxiet*" OR "anxious*" OR "mood disorder*" OR "psychological distress" OR "mental disorder*" OR ("mental*" adj3 ("ill*" OR "unwell")) AND "subthreshold" OR "subclinical" OR "subsyndromal" OR "sub-threshold" OR "sub-clinical" OR "sub-syndromal" OR "subsyndrome" OR "subcase" OR "sub-case".


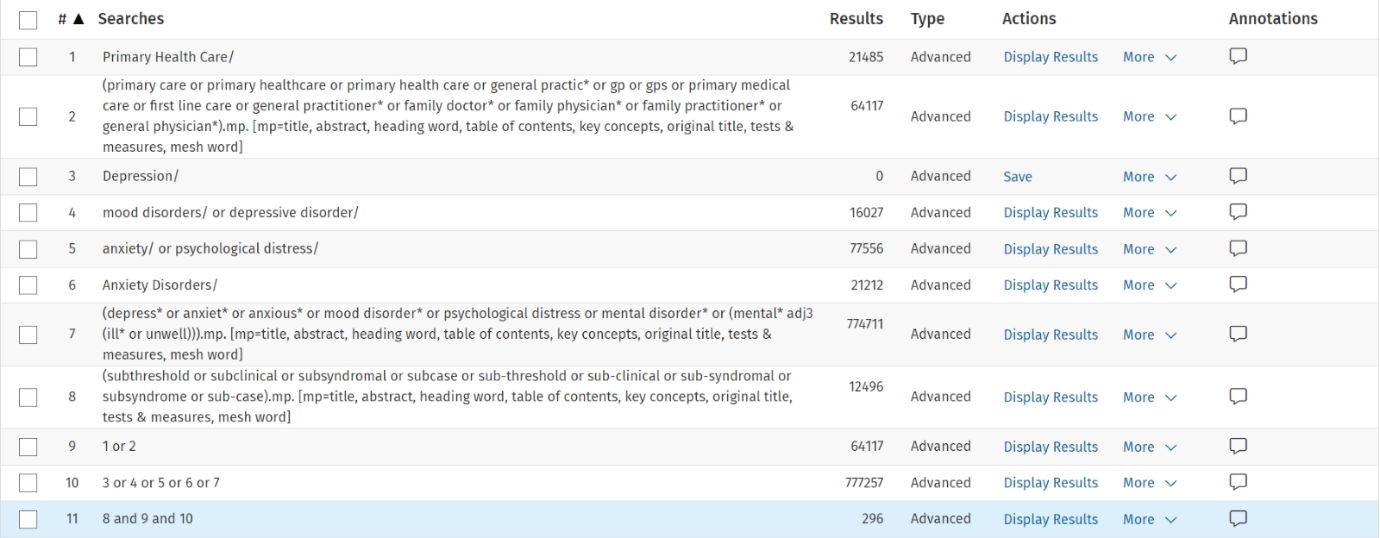


MeSH headings of "Primary Health Care", "Depression", "mood disorders", "depressive disorder", "anxiety", "psychological distress" and "anxiety disorders".
