# Supplementary material for: The Identification and Management of Subthreshold Depression and Anxiety in Primary Care for People With Long-Term Conditions
Source: Depress Anxiety. 2025 Mar 6;2025:9497509. doi: 10.1155/da/9497509 (PMC11987070; doi:10.1155/da/9497509)
Supplement: Supporting Information 2 — The data extraction form used to collect data relevant to the review questions and objectives is outlined in Online File 2. [file 9497509.f2.docx]

**Online File 2 – Data extraction form**

| **Author/Year** | **Title** | **Objectives** | **Study Info** | **Condition** | **Identification** | **Intervention** | **Results and Conclusions** |
| --- | --- | --- | --- | --- | --- | --- | --- |
|  |  |  |  |  |  |  |  |
|  |  |  |  |  |  |  |  |
|  |  |  |  |  |  |  |  |
|  |  |  |  |  |  |  |  |
